# Plasmalogen biosynthesis is spatiotemporally regulated by sensing plasmalogens in the inner leaflet of plasma membranes

**DOI:** 10.1038/srep43936

**Published:** 2017-03-08

**Authors:** Masanori Honsho, Yuichi Abe, Yukio Fujiki

**Affiliations:** 1 Medical Institute of Bioregulation, Kyushu University, 3-1-1 Maidashi, Higashi-ku, Fukuoka 812-8582, Japan

## Abstract

Alkenyl ether phospholipids are a major sub-class of ethanolamine- and choline-phospholipids in which a long chain fatty alcohol is attached at the *sn*-1 position through a vinyl ether bond. Biosynthesis of ethanolamine-containing alkenyl ether phospholipids, plasmalogens, is regulated by modulating the stability of fatty acyl-CoA reductase 1 (Far1) in a manner dependent on the level of cellular plasmalogens. However, precise molecular mechanisms underlying the regulation of plasmalogen synthesis remain poorly understood. Here we show that degradation of Far1 is accelerated by inhibiting dynamin-, Src kinase-, or flotillin-1-mediated endocytosis without increasing the cellular level of plasmalogens. By contrast, Far1 is stabilized by sequestering cholesterol with nystatin. Moreover, abrogation of the asymmetric distribution of plasmalogens in the plasma membrane by reducing the expression of *CDC50A* encoding a β-subunit of flippase elevates the expression level of Far1 and plasmalogen synthesis without reducing the total cellular level of plasmalogens. Together, these results support a model that plasmalogens localised in the inner leaflet of the plasma membranes are sensed for plasmalogen homeostasis in cells, thereby suggesting that plasmalogen synthesis is spatiotemporally regulated by monitoring cellular level of plasmalogens.

Alkenyl ether phospholipids are a subclass of glycerophospholipids characterized by a vinyl ether linkage of fatty alcohols to the *sn*-1 position of the glycerol backbone. Synthesis of ethanolamine (Etn)-containing alkenyl ether phospholipids (plasmalogens) is initiated in peroxisomes and completed in the endoplasmic reticulum (ER) through seven-step reactions^1^. Thereafter, plasmalogens are transported to the post-Golgi compartments mainly via a non-secretory pathway, in a manner dependent on the cellular ATP level^2^. The initial two steps of plasmalogen synthesis are catalyzed by peroxisomal matrix enzymes, dihydroxyacetonephosphate acyltransferase (Dhapat) and alkylglycerone phosphate synthase (Agps), in which 1-alkyl-dihydroxyacetonephosphate (DHAP) is generated by replacing the acyl chain of 1-acyl-DHAP with a long chain fatty alcohol that is synthesized by fatty acyl-CoA reductase 1 (Far1), a peroxisomal C-tail anchored protein^3^,^4^,^5^. Deficiency of plasmalogen synthesis evoked by dysfunction of peroxisomal enzymes essential for the plasmalogen synthesis causes rhizomelic chondrodysplasia punctata (RCDP), a fatal genetic disease^6^,^7^,^8^. Plasmalogen deficiency is also found in peroxisome biogenesis disorders (PBDs) where plasmalogens level is severely reduced in several organs such as brain, heart, and kidney from patients with PBDs^9^, implying that *de novo* synthesis of plasmalogens plays a pivotal role in the tissue plasmalogen homeostasis.

We earlier showed that plasmalogen synthesis is regulated by modulating the stability of Far1 in response to the cellular levels of plasmalogens^4^,^5^. Elevation of plasmalogens is found in tumour^10^,^11^,^12^, whereas plasmalogens are reduced in the patients with several diseases including sporadic Alzheimer’s disease and Pelizaeus-Merzbacher disease^13^,^14^,^15^. In addition, the cellular plasmalogen level plays an important role in cholesterol homeostasis at multiple steps including cholesterol synthesis^16^, esterification of cholesterol^17^, high-density lipoprotein (HDL)-mediated cholesterol efflux^18^, internalization of cholesterol^19^, and distribution of cholesterol^20^. Given these findings, plasmalogen synthesis must be tightly regulated to maintain the homeostasis of plasmalogens by sensing the cellular level of plasmalogens. However, the mechanisms of regulation in plasmalogen synthesis remain undefined. To address this issue, we investigated how cells sense the cellular level of plasmalogens. The results suggest that plasmalogens localised in the inner leaflet of the plasma membrane are sensed, of which signal is transferred to peroxisomes to modulate the stability of Far1.

## Results

### Plasmalogen-induced degradation of Far1 in wild-type cells

**Figure 1:**
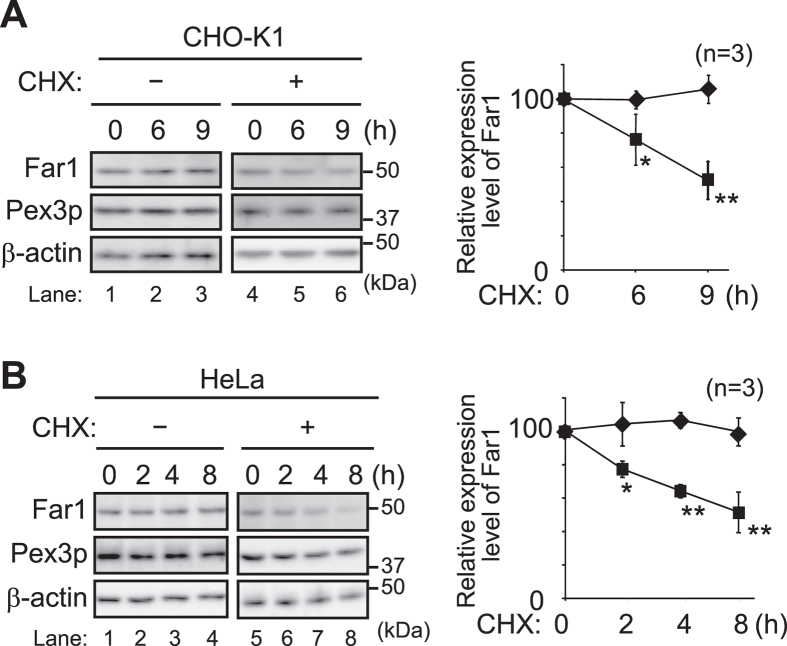
Plasmalogen-iduced degradation of Far1 in wild-type cells. CHO-K1 (**A**) and HeLa (**B**) cells were cultured in the presence (+) or absence (−) of CHX for the indicated time. Expression level of Far1 at each time point in the absence (*diamond*) or presence (*square*) of CHX was assessed. Peroxisomal membrane protein Pex3p and *β–*actin for a loading control were detected with respective specific antibodies (*left panels*). Molecular masses were indicated on the right, using the migration of standard mass markers. Relative amounts of Far1 at each time point were represented by taking as 100 those at the time point of CHX addition in respective cells (*right panels*). *p < 0.05, **p < 0.01; Student’s *t* test compared with the expression level of Far1 at each time point in the absence of CHX. Data of unprocessed original blots are available in Supplementary Fig. S2.

Plasmalogen biosynthesis is regulated by modulating the stability of Far1 in response to the cellular level of plasmalogens^4^. We earlier showed that restoration of plasmalogens in the plasmalogen-deficient cell mutants such as *agps* ZPEG251 or elevation of plasmalogens in wild-type cells enhances the degradation of Far1^4^,^5^. In addition, the expression level of Far1 in CHO-K1 and HeLa cells was reduced in the presence of cycloheximide (CHX) (Fig. 1), whereas degradation of Far1 is suppressed in ZPEG251^4^, implying that Far1 is degraded by the turnover rate to maintain the homeostasis of plasmalogens through the constitutive sensing of cellular plasmalogens in wild-type cells (hereafter called plasmalogen-induced degradation of Far1).

### Dynamin-mediated endocytosis is involved in the regulation of plasmalogen synthesis

**Figure 2:**
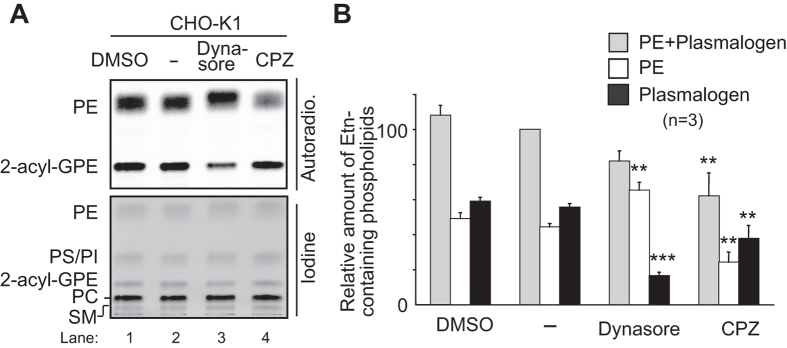
Dynasore reduces plasmalogen synthesis. (**A**) CHO-K1 cells were incubated with DMSO, Dynasore, or CPZ for 30 min and further metabolically labelled with ^14^C-Etn for 5 h in the absence (−) and presence of each inhibitor. The biosynthesis of PE and plasmalogen (2-acyl-GPE) were detected with a FLA-5000 imaging analyser (*upper panel*). Total cellular level of phospholipids including PE, PS, phosphatidylinositol (PI), plasmalogens, phosphatidylcholine (PC), and SM were detected with iodine vapor (*lower panel*). Note that biosynthesis of plasmalogens was specifically reduced in the presence of Dynasore. (**B**) Biosynthesis of PE (*open bar*), plasmalogens (*dark grey bar*), PE plus plasmalogens (*light grey bar*) was represented by taking the amounts of PE plus plasmalogens as 100 in mock-treated (−) CHO-K1. Statistical analysis was performed by one-way ANOVA with Dunnett’s post hoc test as compared with the synthesis of respective lipids in mock-treated cells. *p < 0.05, **p < 0.01, and ***p < 0.001.

**Figure 3:**
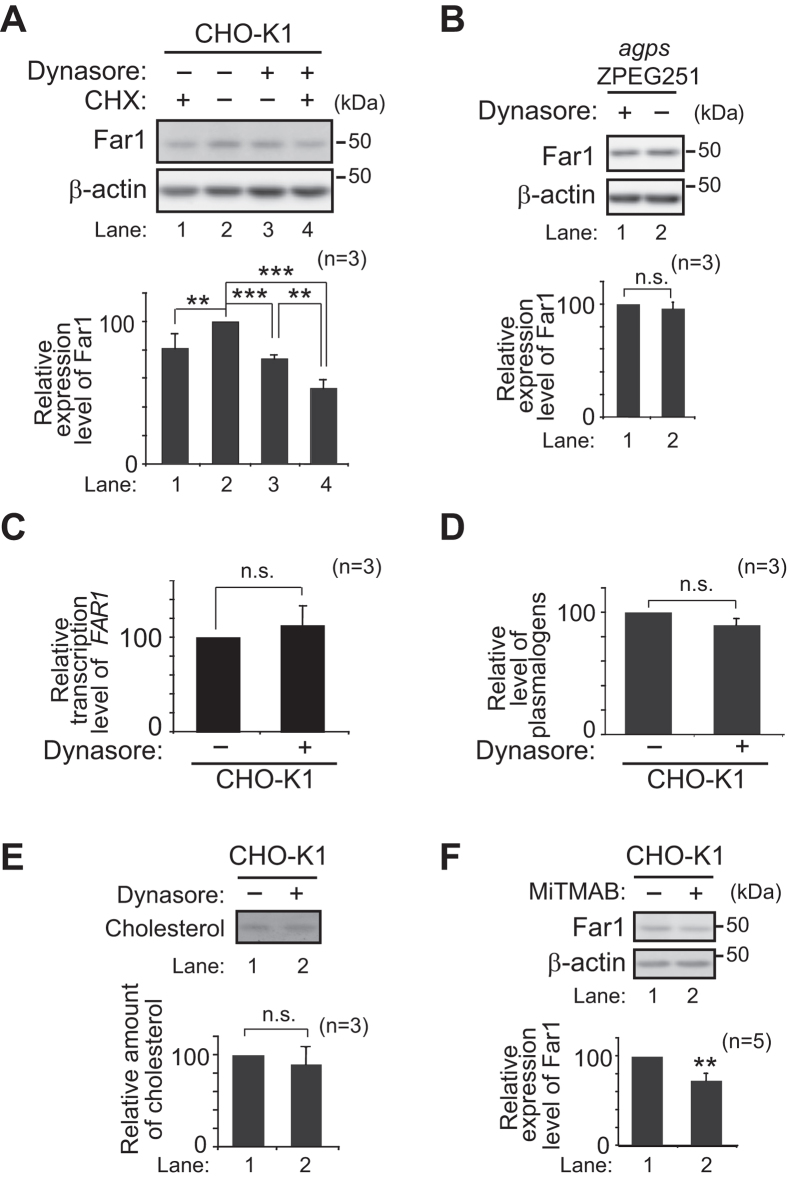
Expression level of Far1 is reduced in the presence of Dynasore. (**A**) CHO-K1 cells were cultured for 5 h in the presence (+) of CHX (lane 1), Dynasore (lane 3), or CHX plus Dynasore (lane 4) and were assessed for the expression level of Far1 (*upper panel*). *β–*actin was used as a loading control. Relative expression levels of Far1 were represented by taking those as 100 in mock-treated cells (lane 2, *lower panel*). Note that Far1 expression was reduced in the presence of Dynasore in CHO-K1 cells (lane 3). **p < 0.01 and ***p < 0.001; two-way ANOVA with Tukey-Kramer post hoc test. (**B**) *agps* ZPEG251 was cultured for 5 h in the presence (+) or absence (−) of Dynasore and were assessed for the expression level of Far1 (*upper panel*). Relative expression levels of Far1 were represented by taking those as 100 in mock-treated cell (*lower panel*). n.s., not significant; *t* test. (**C**) Expression level of *FAR1* relative to *β–Actin* was determined by real-time PCR using total RNA, and represented by taking as 100 that in CHO-K1 (−). n.s., not significant; *t* test. (**D**) Total cellular plasmalogens were analysed by LC-ESI-MS/MS and represented by taking that as 100 in CHO-K1 (−). n.s., not significant; *t* test. (**E**) Lipids were extracted from cells cultured in the absence (−) or presence (+) of Dynasore and assessed by TLC (*upper panel*) as described in Materials and Methods. Cholesterol was detected with iodine vapor and quantified (*lower panel*) using an image analysis software (Multi Gauge). n.s., not significant; *t* test. (**F**) CHO-K1 cells were cultured for 5 h in the presence (+) or absence (−) of MiTMAB and assessed for the expression level of Far1 (*upper panel*). Relative expression levels of Far1 were represented by taking those as 100 in mock-treated cells (*lower panel*). **p < 0.01; *t* test. Data of unprocessed original blots are available in Supplementary Fig. S2.

Plasmalogens are transported from the ER to the post-Golgi compartments in a manner dependent on ATP, but independent on secretory pathway^2^. However, plasmalogens are not localised to peroxisomes^2^,^21^,^22^. Therefore, we suspected that plasmalogens in either ER or the post-Golgi compartments including plasma membrane are sensed for the regulation of plasmalogen synthesis. To test if plasmalogens in the plasma membrane are sensed, we attempted to inhibit endocytic pathways with Dynasore, an inhibitor of dynamin^23^. Dynamin plays a crucial step in the fission of newly formed vesicles in several endocytic pathways, including clathrin-mediated and clathrin-independent endocytic processes such as caveolin- as well as clathrin- and caveolin-independent pathways^24^. When cells were incubated with ^14^C-Etn in the presence of Dynasore, plasmalogen synthesis was reduced to about 30% of that in mock-treated cells, whereas synthesis of phosphatidylethanolamine (PE) was elevated (Fig. 2, lanes 2 and 3). These results are similar to the synthesis of plasmalogens and PE in CHO cell mutants defective in early step(s) of plasmalogen synthesis including *DHAPAT*-defective NRel-4^25^ and *AGPS*-deficient ZPEG251^2^ and NZel-1^26^. In contrast, treatment with chlorpromazine (CPZ), an inhibitor of clathrin-dependent endocytosis and macropinocytosis^27^, lowered the synthesis of both plasmalogens and PE in a similar fashion, indicating that the plasmalogen synthesis was not specifically reduced by CPZ, rather incorporation of ^14^C-Etn and/or the common pathway(s) for the synthesis of both plasmalogens and PE were suppressed (Fig. 2, lanes 2 and 4). However, expression and peroxisomal localisation of Dhapat and Agps were not altered in the presence of Dynasore (Supplementary Fig. S1A,B). We next assessed the expression of Far1, a rate-limiting enzyme of plasmalogen synthesis^4^,^5^. Protein level of Far1 was reduced upon the treatment with Dynasore in CHO-K1 (Fig. 3A, lanes 2 and 3), which was further decreased in the presence of CHX (Fig. 3A, lane 4). In contrast, expression of Far1 was not altered by treatment with Dynasore in *agps* ZPEG251 (Fig. 3B), where accumulation of transferrin (Tf) in recycling endosomes was inhibited with Dynasore as reported^23^ (Supplementary Fig. S1C, *lower panel*). Transcription of *FAR1* in CHO-K1 cells was not reduced in the presence of Dynasore (Fig. 3C). Together, these results suggest that plasmalogen-induced degradation of Far1 is augmented by Dynasore. However, total cellular level of plasmalogens was not elevated in the presence of Dynasore (Fig. 3D).

Dynasore inhibits GTPase activity of dynamin-like protein 1 (Dlp1) both *in vitro*^23^ and *in vivo*^28^,^29^. Therefore, we investigated whether Dynasore-mediated inactivation of Dlp1 induces degradation of Far1 using *dlp1* CHO mutant ZP121^30^. Consistent with the partial defect of plasmalogen synthesis in ZP121^30^, expression level of Far1 in ZP121 was higher than that in CHO-K1 cells (Supplementary Fig. S1D), implying that inactivation of Dlp1 is not the primary cause for the reduction of the expression level of Far1 upon the treatment with Dynasore in CHO-K1 cells. Dynasore also reduces cellular cholesterol and disrupts rafts^31^. However, we did not observe the reduction of cholesterol when CHO-K1 cells were incubated with Dynasore (Fig. 3E). Finally, we verified the expression level of Far1 with myristyl trimethyl ammonium bromide (MiTMAB), an inhibitor that inhibits GTPase activity of dynamin by disrupting the interaction between pleckstrin homologydomain of dynamin and phospholipids^32^, and found that the expression level of Far1 was reduced to about 70% of that in mock-treated CHO-K1 cells upon the treatment with MiTMAB (Fig. 3F). Taken together, these results suggest that inhibition of dynamin-mediated endocytic pathways most likely accelerates plasmalogen-induced degradation of Far1.

### Knockdown of flotillin-1 enhances plasmalogen-induced degradation of Far1

**Figure 4:**
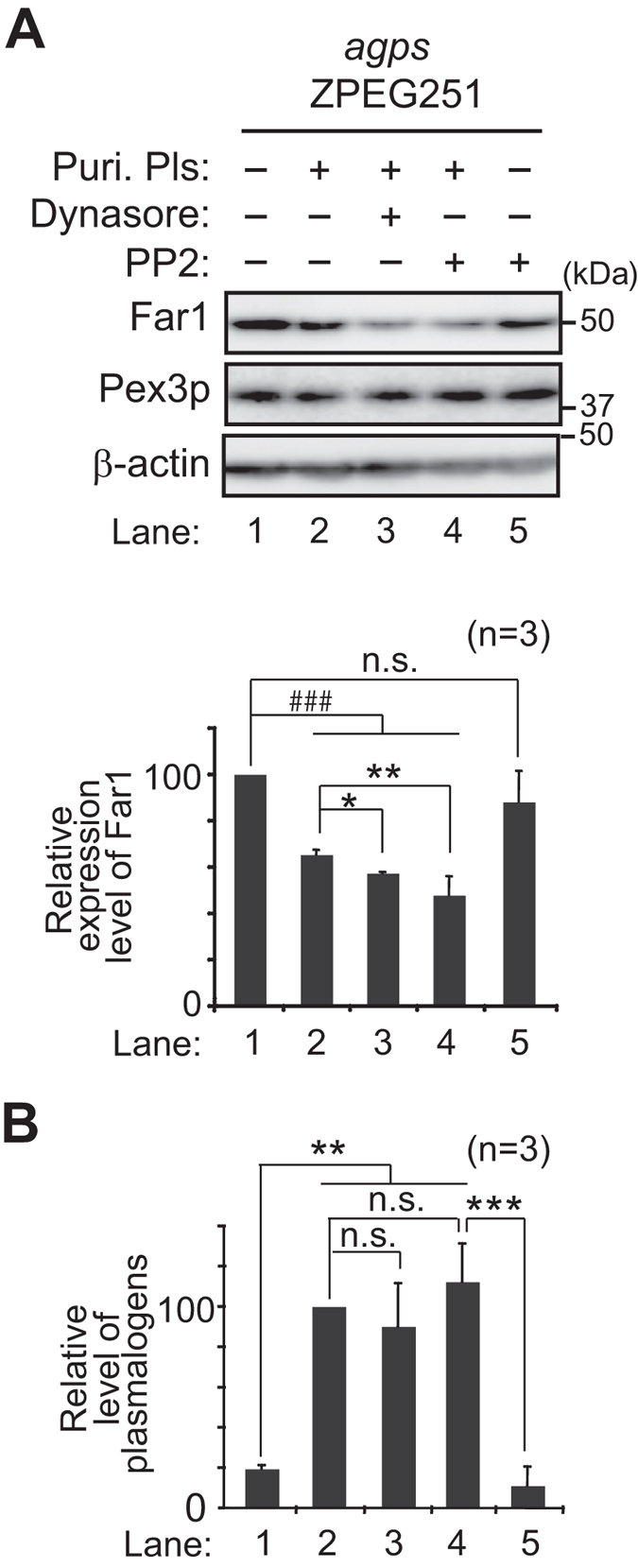
Src family kinase inhibitor PP2 induces degradation of Far1. (**A**) *agps* ZPEG251 was cultured in the presence (lanes 2–4) or absence (lanes 1 and 5) of purified plasmalogens (puri. Pls) for 6 h, and further cultured in the presence of Dynasore (lane 3) or PP2 (lanes 4 and 5) for 5 h. Expression levels of Far1 were assessed by immunoblotting (*upper panel*) and represented (*lower panel*) by taking as 100 that in ZPEG251 (lane 1). ^###^*p* < 0.001; one-way ANOVA with Dunnett’s post hoc test as compared with mock-treated cells. n.s., not significant. *p < 0.05 and **p < 0.01; one-way ANOVA with Dunnett’s post hoc test as compared with ZPEG251 treated with purified plasmalogens (lane 2). (**B**) Cellular plasmalogens were analysed by LC-ESI-MS/MS and represented by taking as 100 that in ZPEG251 cultured in the presence of purified plasmalogens (lane 2). **p < 0.01 and ***p < 0.001; one-way ANOVA with Tukey post hoc test as compared with ZPEG251. n.s., not significant. Data of unprocessed original blots are available in Supplementary Fig. S2.

**Figure 5:**
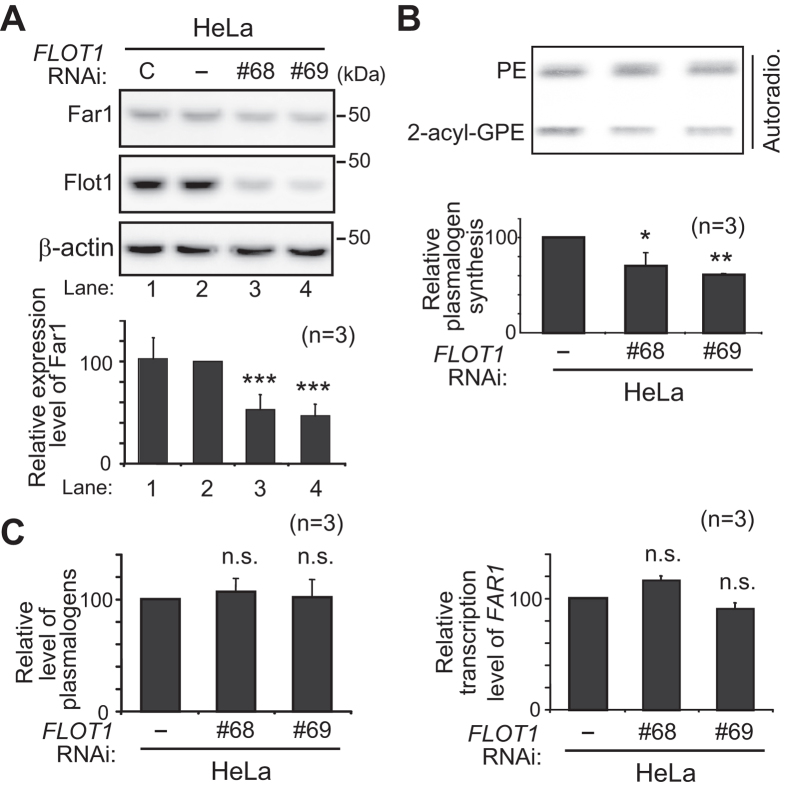
Knockdown of *FLOT1* lowers Far1 expression. (**A**) Expression levels of Far1 and Flot1 were assessed in HeLa cells transfected with control dsRNA (lane 1) or two different dsRNAs (lanes 3 and 4) against *FLOT1*. Expression level of Far1 was represented by taking as 100 that in mock-treated cell (*lower panel*). (**B**) Plasmalogen synthesis was assessed in mock-treated (−) or *FLOT1*-knocked down (#68 and #69) HeLa cells by metabolically labelling with ^14^C-Etn for 2 h (*upper panel*). Biosynthesis of plasmalogens was represented by taking as 100 that in mock-treated cells (*lower panel).* *p < 0.05, **p < 0.01, and ***p < 0.001; one-way ANOVA with Dunnett’s post hoc test as compared with mock-treated cells. (**C**) *Left panel*, total cellular level of plasmalogens was represented by taking as 100 that in mock-treated cells. *Right panel*, expression level of *FAR1* relative to ribosomal protein 3 (*RPL3)*^63^ was determined by real-time PCR using total RNA, and represented by taking as 100 that in HeLa cells (−). n.s., not significant; one-way ANOVA with Dunnett’s post hoc test as compared with mock-treated cells. Data of unprocessed original blots are available in Supplementary Fig. S2.

**Figure 6:**
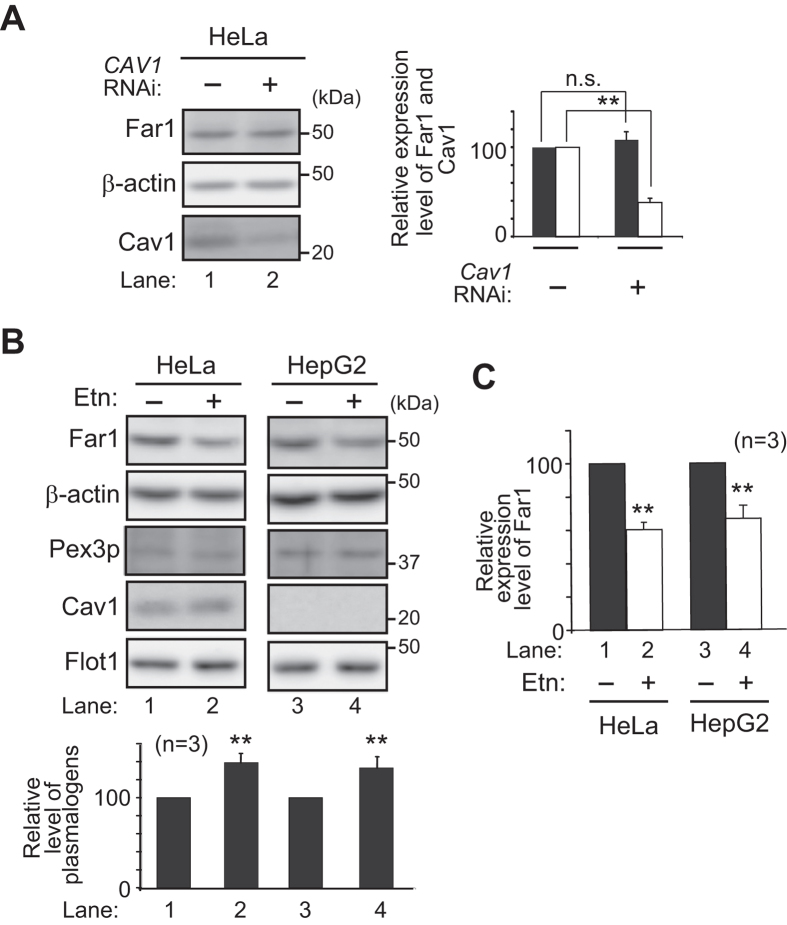
Far1 is degraded in the absence of caveolae. (**A**) Expression levels of Far1 (*solid bar*) and Cav1 (*open bar*) were assessed in HeLa cells transfected with dsRNAs against *CAV1. β–*actin was used as a loading control. Expression level of Far1 was represented by taking as 100 that in mock-treated cell (*right panel*). **p < 0.01; *t* test *versus* mock-treated cells. n.s., not significant. (**B**) HeLa and HepG2 were cultured in the presence of Etn (5 μM) for 48 h and assessed for expression level of Far1, *β–*actin, Pex3p, Cav1, and Flo1. Note that Cav1 is not expressed in HepG2 cells^35^. Cellular plasmalogens were analysed by LC-ESI-MS/MS and represented by taking those as 100 in mock-treated respective cells. **p < 0.01; *t* test *versus* mock-treated respective cells. (**C**) Expression level of Far1 was represented by taking those as 100 in mock-treated respective cells. **p < 0.01; *t* test *versus* mock-treated respective cells. Data of unprocessed original blots are available in Supplementary Fig. S2.

We further investigated whether inhibition of caveolin- or flotillin-1 (Flot1)-mediated endocytosis enhances plasmalogen-induced degradation of Far1. To this end, plasmalogen-deficient *agps* ZPEG251 cells were cultured with purified plasmalogens in the presence of PP2, an inhibitor specific for Src-kinase essential for both types of caveolin-dependent and Flot1-mediated endocytosis^33^,^34^. Expression of Far1 was lowered in ZPEG251 cells upon culturing with purified plasmalogens as compared to that in untreated ZPEG251, which was further reduced in the presence of Dynasore (Fig. 4A, lanes 1–3), confirming that inhibition of dynamin-mediated endocytosis induces degradation of Far1 (Fig. 3A, lanes 2 and 3). Protein level of Far1 was similarly reduced in ZPEG251 in the presence of PP2 and purified plasmalogens, whereas PP2 alone did not affect the expression level of Far1 (Fig. 4A, lanes 4 and 5). Under these conditions, total amount of cellular plasmalogens was not altered by the treatment with Dynasore and PP2 as compared with those in ZPEG251 cells cultured with purified plasmalogens (Fig. 4B). Furthermore, protein level of Far1 was reduced by nearly 50% upon knocking down *FLOT1* using two independent double-stranded RNAs (dsRNAs) (Fig. 5A, *lower panel*), where Flot1 protein level was lowered to about 20% of that in mock-treated cells (Fig. 5A, *upper panel*). Consistent with these results, the knockdown of *FLOT1* reduced the plasmalogen synthesis to about 50% level of that in HeLa cells (Fig. 5B), whereas transcription of *FAR1* and total cellular plasmalogen level were not altered (Fig. 5C). In contrast, protein level of Far1 was not altered under the more than 60% reduction of caveolin1 (Cav1) expression in HeLa cells treated with *CAV1* RNAi (Fig. 6A). Next, we examined whether elevation of plasmalogens stimulates degradation of Far1 in HepG2 cells, a cell line that shows no detectable caveolins and caveolae^35^ but expresses Flot1 (Fig. 6B). When HeLa cells were cultured in the presence of Etn, cellular plasmalogen level was increased about 1.5-fold as compared to that in mock-treated cells^16^, thereby stimulating the degradation of Far1 by sensing the elevated level of plasmalogens (Fig. 6B,C, lanes 1 and 2)^4^,^5^. Similar extent of the degradation of Far1 was observed in HepG2 cells as the plasmalogen level was increased by supplementation of Etn (Fig. 6B,C, lanes 3 and 4), implying that plasmalogen-induced degradation of Far1 is not abrogated under the suppressed caveolin-mediated endocytosis. Together, these results suggest that inhibition of Flot1-mediated endocytosis most likely enhances plasmalogen-induced degradation of Far1.

### Sensing of plasmalogens in the inner leaflet of plasma membrane

**Figure 7:**
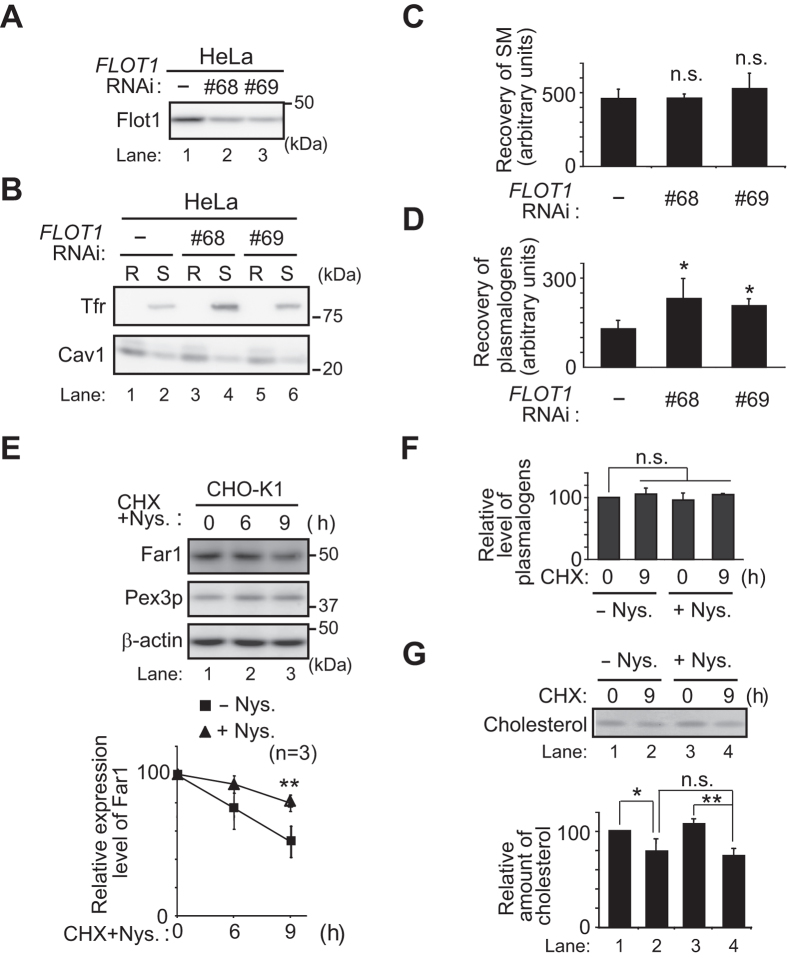
Knockdown of *FLOT1* elevates the amount of plasmalogens and nystatin inhibits degradation of Far1. (**A**) HeLa cells were transfected with two different dsRNAs (lanes 2 and 3) against *FLOT1* and expression of Flot1 was assessed. (**B**) DRMs (R) and detergent-soluble (S) fractions were prepared as described in Materials and Methods and distribution of Cav1 and Tfr was assessed using respective antibodies. (**C**,**D**) Amount of SM (**C**) and plasmalogens (**D**) in DRMs prepared as in (**B**) was determined by LC-ESI-MS/MS and represented by arbitrary units. *p < 0.05; one-way ANOVA with Dunnett’s post hoc test *versus* mock-treated HeLa cells. n.s., not significant. (**E**) CHO-K1 cells were cultured for 1 h with nystatin (Nys.), a cholesterol-chelating agent, further incubated for the indicated time in the presence of CHX, and assessed for the expression of Far1 at each time point (*upper panel*). *β–*actin was used as a loading control. Relative amounts of Far1 in the presence (*triangle*) of nystatin at each time point were represented by taking as 100 that at the time point of CHX addition. The relative expression level of Far1 in the absence (*square*) of nystatin was from Fig. 1A (*left panels*). **p < 0.01; *t* test compared with the expression level of Far1 at 9 h in the absence of nystatin. (**F**) Cellular plasmalogens in CHO-K1 cells cultured in the absence (−Nys.) or presence (+Nys.) of nystatin were analysed by LC-ESI-MS/MS and their relative level was represented by taking as 100 that at (0 h, -Nys.). n.s., not significant by two-way ANOVA. (**G**) Lipids were extracted from cells cultured as in (**F**). Cholesterol was detected as described in Fig. 3E. Relative amount of cholesterol is represented by taking as 100 that at (0 h, -Nys.). *p < 0.05 and **p < 0.01; two-way ANOVA with Tukey post hoc test. n.s., not significant. Note that cellular cholesterol was reduced by the treatment of CHX but not with nystatin.

Given the findings that plasmalogen-induced degradation of Far1 is enhanced by the knockdown of Flot1 without elevating total cellular plasmalogen level (Fig. 5C), we suspected that the degradation of Far1 is accelerated by sensing plasmalogens locally enriched in the plasma membrane upon inhibiting Flot1-mediated endocytosis. In this hypothesis, elevation of plasmalogens in lipid rafts might be a prerequisite for the sensing because plasmalogens are enriched in lipid rafts^2^,^36^ and knocking down of *FLOT1* inhibits the endocytosis of raft-associated proteins such as CD59, i.e. a glycosylphosphatidylinositol (GPI)-linked protein, and a cholera toxin B subunit in HeLa cells^37^. Therefore, we verified the amount of plasmalogens and sphingomyelin (SM) in the detergent-resistant membranes (DRMs) in *FLOT1*-knocked down HeLa cells (Fig. 7). When HeLa cells were solubilized with Triton X-100 on ice and separated into DRMs and Triton X-100 soluble fractions by floatation on a 30% iodixanol gradient, Cav1 was mostly recovered in DRMs, while Tf receptor (Tfr), a non-raft protein, was completely solubilized by Triton X-100 as reported^2^ (Fig. 7B, lanes 1 and 2). In *FLOT1*-knocked down HeLa cells, Cav1 and SM, not Tfr, were recovered in DRMs similar to those in mock-treated HeLa cells where the transfection of one of the dsRNAs against *FLOT1* induced protein level of Tfr (Fig. 7B,C). However, amount of plasmalogens in DRMs prepared from *FLOT1*-knocked down HeLa cells was about 1.5-fold higher than that in mock-treated cells (Fig. 7D), suggesting that plasmalogens are locally enriched in lipid rafts upon reducing the expression of Flot1. We further examined if plasmalogens in the plasma membrane are sensed by sequestering cholesterol in the plasma membrane. To this end, CHO-K1 cells were treated with nystatin, a cholesterol-chelating agent, inducing distortion of the structure and function of cholesterol-rich membrane domain^38^ and assessed for the degradation of Far1 in the presence of CHX. Degradation of Far1 was significantly suppressed by the treatment with nystatin (Fig. 7E) without reducing total cellular level of plasmalogens and cholesterol (Fig. 7F,G). We interpreted these results to mean that the plasmalogen-induced degradation of Far1 is most likely retarded by interfering with the sensing steps of plasmalogens localised in the plasma membrane.

**Figure 8:**
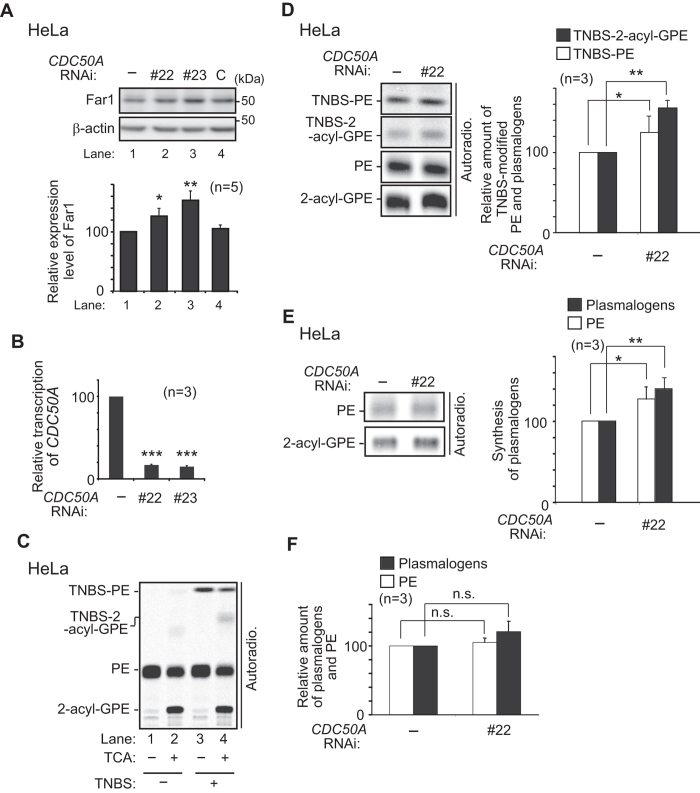
Elevation of plasmalogens localised at the outer leaflet of plasma membrane by *CDC50A* knockdown increases the expression of Far1. (**A**) Expression level of Far1 was assessed in mock-treated HeLa cells (−) or cells transfected with control dsRNA (**C**) or two different dsRNAs (#22 and #23) against *CDC50A (upper panel*). *β–*actin was used as a loading control. Expression level of Far1 was represented by taking as 100 that in mock-treated cell (*lower panel*). *p < 0.05 and **p < 0.01; one-way ANOVA with Dunnett’s post hoc test *versus* mock-treated cells. (**B**) HeLa cells were cultured as described in (**A**). Expression level of *CDC50A* relative to *RPL3*^63^ was determined by real-time PCR using total cell RNA. ***p < 0.001; one-way ANOVA with Dunnett’s post hoc test *versus* mock-transfected cells (−). (**C**) HeLa cells were cultured in the presence of ^14^C-Etn for 18 h and further incubated with (+) or without (−) TNBS. Total cellular lipids were extracted from aliquots of equal amounts of cell proteins before (−) or after (+) treatment with TCA and analysed by TLC. (**D**) PE and plasmalogens localised at the outer leaflet of plasma membrane were verified as in (**C**) in mock-transfected HeLa cells (−) or *CDC50A*-knocked down cells by transfection with dsRNA (#22) (*left panel*). Relative amounts of TNBS-modified PE (TNBS-PE, *open bar*) and plasmalogens (TNBS-2-acyl-GPE, *solid bar*) were represented by taking as 100 those in mock-transfected cells (*right panel).* *p < 0.05 and **p < 0.01; *t* test *versus* mock-transfected cells. (**E**) Plasmalogen synthesis was assessed in mock-transfected (−) or *CDC50A*-knocked down (#22) HeLa cells by metabolically labelling with ^14^C-Etn for 5 h (*left panel*). Biosynthesis of PE (*open bar*) and plasmalogens (*solid bar*) was represented by taking as 100 those in mock-transfected cells (*right panel).* *p < 0.05 and **p < 0.01; *t* test *versus* mock-transfected cells. (**F**) Relative level of PE (*open bar*) and plasmalogens (*solid bar*) in *CDC50A*-knocked down (#22) HeLa cells was represented by taking as 100 those in mock-transfected cells. n.s., not significant; *t* test *versus* mock-transfected cells. Data of unprocessed original blots are available in Supplementary Fig. S2.

Finally, we assessed if plasmalogens located in the inner leaflet of the plasma membrane are sensed. It is known that aminophospholipids such as phosphatidylserine (PS) and PE are concentrated in the inner leaflet of the plasma membrane, where type-IV P-type ATPases (P4-ATPases) function in the phospholipid transfer from the outer leaflet to the inner leaflet^39^. Since plasmalogens are also shown located in the inner leaflet of plasma membrane in red blood cells and myelin^40^,^41^, we verified the protein level of Far1 by reducing the expression of *CDC50A* encoding a β-subunit of P4-ATPases that is essential for exiting of most of P4-ATPases from the ER by forming a heterooligomer^42^,^43^. Knockdown of *CDC50A* using two independent dsRNAs elevated the protein level of Far1 (Fig. 8A,B). We also determined distribution of plasmalogens at the outer leaflet with 2,4,6-trinitrobenzene sulfonic acid (TNBS), a membrane impermeable amine-reactive reagent^44^. TNBS-modified PE was detected with a higher mobility on TLC than PE. Upon the trichloroacetic acid (TCA)-treatment, by which the vinyl ether linkage of plasmalogens is hydrolysed, thereby giving rise to two more slower-migrating bands each corresponding to TNBS-plasmalogens (TNBS-2-acyl-GPE) and unmodified plasmalogens (2-acyl-GPE) (Fig. 8C), where TNBS-plasmalogens and TNBS-PE represented 3.7 ± 0.2% and 6.8 ± 0.5%, respectively, of total plasmalogens and PE (Fig. 8C, lane 4). Knockdown of *CDC50A* elevated the levels of TNBS-plasmalogens and TNBS-PE (Fig. 8D) and augmented the synthesis of plasmalogen and PE (Fig. 8E) where total amount of PE and plasmalogens was not significantly increased (Fig. 8F). Collectively, these results suggest that plasmalogens located in the inner leaflet of plasma membrane are sensed for detection of the cellular level of plasmalogens.

## Discussion

Plasmalogen synthesis is regulated by a feedback mechanism in a manner dependent on the cellular level of plasmalogens^4^,^5^. In the present study, we showed that plasmalogen-induced degradation of Far1 is enhanced by the inhibition of dynamin-, Src kinase-, and Flot1-mediated endocytosis (Figs 3,4 and 5), whereas perturbation of inner leaflet localisation of plasmalogens or sequestering of cholesterol in the plasma membrane suppressed the degradation of Far1 without altering total cellular level of plasmalogens (Figs 7 and 8). Together, these results suggest that plasmalogens localised in the inner leaflet of the plasma membrane is critical for sensing of the cellular level of plasmalogens.

Flot1 is shown to be enriched in lipid rafts of the plasma membrane^37^,^45^ and its association with lipid rafts in *DHAPAT*-knockout mice is reduced^46^, suggesting that the association of Flot1 with lipid rafts is involved in sensing of plasmalogens. If so, knocking down of *FLOT1* would abrogate the sensing system for plasmalogens, stabilize Far1, and elevate plasmalogen synthesis. However, our data showed the converse that knockdown of *FLOT1* reduced the expression of Far1 and plasmalogen synthesis, suggesting that another unidentified protein, if any, monitors the cellular level of plasmalogens.

Flot1 is involved in signaling pathway, cell adhesion, and membrane trafficking^47^. Our findings that the inhibition of dynamin or Src-kinase reduce the expression of Far1 and knocking down of *FLOT1* elevates the plasmalogen level in DRMs (Fig. 7D) enriched in cholesterol and SM, both most abundant in the plasma membrane^48^, suggest that inhibition of the Flot1-mediated endocytic pathway stimulates the degradation of Far1 by increasing the plasmalogen level in the plasma membrane. Based on these findings, we propose that newly synthesized plasmalogens are transported from the ER to the plasma membrane and dynamically endocytosed, rather than being statically localised in plasma membrane. Accordingly, Flot1-mediated endocytosis plays an important role in the plasmalogen homeostasis, while caveolae is less likely involved in the plasmalogen-dependent degradation of Far1 (Fig. 6). Noteworthily, caveolae is not found in neuron^49^,^50^ and lymphocytes^51^. In contrast, Flot1 is expressed in several cell types including neuron^52^,^53^ and lymphocytes^53^,^54^. Together, our findings suggest that Flot1-mediaed endocytosis plays a crucial role in the plasmalogen homeostasis in most tissues. At the next steps toward elucidation of the flotillin-mediated endocytic pathway in plasmalogen homeostasis, flotillin-2 (Flot2) may need to be verified since Flot2 that is 47% identical to Flot1 in the primary structure^55^ functions together with Flot1 to generate flotillin microdomains by binding to plasma membrane inner leaflet via SPFH (stomatin, prohibitin, flotillin, Hflk/C) domain and acylation of the cysteine residues in its N-terminal hydrophobic stretch^56^.

The physiological consequence of the enrichment of plasmalogens in lipid rafts has not been defined. Plasmalogens do not seem to be a structurally essential constituent for the formation of lipid rafts^2^. The interference with cholesterol-mediated assembly and function of the membrane by nystatin significantly reduces degradation of Far1 (Fig. 7E). Nystatin does not cross the bilayer and enter the cells^57^, supporting a model for sensing of plasmalogens in the plasma membrane. Nystatin disrupts caveolae morphology and inhibits the function of caveolae without altering clathrin-mediated endocytosis^38^. However, the elevated level of plasmalogens was sensed, followed by stimulating degradation of Far1 in caveolae-lacking HepG2 cells (Fig. 6). Therefore, plasmalogens that reside within cholesterol-rich domain of the plasma membrane such as non-caveolae rafts seem to be important for the sensing of plasmalogens. Clearly, more precise manipulation of membrane property or lipid constituent in non-caveolae rafts and identification of the molecule sensing plasmalogens are required for full understanding of the sensing step(s) of plasmalogens.

Plasmalogens are located in the inner leaflet of plasma membrane in red blood cells and myelin, which is shown by using spin-labelled plasmalogens in red blood cells^40^ or by X-ray with mercuric chloride in myelin^41^, respectively. By a combination of TNBS modification and TCA treatment of the extracted lipids, one-step analysis was made feasible in the detection of TNBS-PE, TNBS-plasmalogens, unmodified PE, and plasmalogens (Fig. 8C). In the analysis using HeLa cells, 5.2 ± 0.2% of total Etn-containing phospholipids corresponding to plasmalogens plus PE were modified with TNBS, although the level of TNBS-modified Etn-phospholipids was slightly lower than that obtained from CHO-K1 in G1 phase^44^. Therefore, TNBS-modification assay is also useful and a simple procedure for the detection of outer leaflet-localised plasmalogens. In HeLa cells, about 4% of total plasmalogens were modified by TNBS, less than that of PE (Fig. 8C, lane 4), implying that asymmetric distribution of PE and plasmalogens, both types of phospholipids harboring Etn in their head group, is distinctly regulated. To date, fourteen P4-ATPases are identified in humans and the importance of ATP8A1-mediated PE translocation is assessed in cell migration^58^. We show here that knockdown of *CDC50A* significantly elevates localisation of plasmalogens at cell-surface, giving rise to the increase in plasmalogen synthesis by reducing plasmalogen-induced degradation of Far1, hence implying that P4-ATPase-mediated asymmetric distribution of plasmalogens is important for sensing the plasmalogens. We should await identification of P4-ATPase(s) responsible for the topogenesis of plasmalogens and delineation of the physiological roles of the P4-ATPase(s) in cellular functions.

In summary, plasmalogen biosynthesis is regulated by modulating the stability of Far1 by sensing the plasmalogens localised in the inner leaflet of plasma membrane, where several mechanisms involving Flot1-mediated endocytosis, P4-ATPase-dependent asymmetric distribution of plasmalogens, and recognition of plasmalogens by yet unidentified proteins, if any, coordinately contribute. Subsequently, a signal emerged from sensing the plasmalogen level is spatially transferred to peroxisomes, where the Far1 stability is modulated. Therefore, plasmalogen synthesis is spatiotemporally regulated via several machineries locating in the plasma membrane and peroxisomes.

## Materials and Methods

### Cell culture

HeLa^4^ and HepG2 (a gift from Dr. H. Sumimoto. Kyushu Univ.) were cultured in DMEM supplemented with 10% FBS (Biowest) in 5% CO_2_ and 95% air^4^. Chinese hamster ovary (CHO)-K1and *adaps* ZPEG251^2^ were cultured in Ham’s F-12 medium supplemented with 10% FBS in 5% CO_2_ and 95% air^2^. Cells were cultured in the presence of several types of inhibitors as follows: CHO-K1 cells were cultured in the presence of 80 μM Dynasore (Sigma)^23^, 25 μM CPZ (Sigma), or 30 μM MiTMAB (TOCRIS) for 5 h. ZPEG251 was treated with 10 μM PP2 (Sigma) for 5 h in the presence of purified plasmalogens^4^. CHO-K1 cells were treated with 25 μg/ml nystatin (Sigma) for 1 h and further cultured in the presence of 10 μg/ml cycloheximide (Nacalai Tesque) for the indicated period. Knockdown of *FLOT1, CAV1*, and *CDC50A* was performed by transfecting dsRNAs against respective genes to HeLa cells and the cells were cultured for 3 days. The target sequences of the dsRNA are as follows: human Flotillin-1–68, 5′-GGGCATCAGTGTGGTTAGCTACACT-3′; human Flotillin-1–69, 5′- CGGGAAGCTAAAGCCAAGCAGGAAA-3′ (Invitrogen); human Caveolin1-9504, 5′-CCCTAAACACCTCAACGATTT-3′ (Sigma); human CDC50A-22, 5′-GAGCTATTGCCAACAGCATTT-3′; and human CDC50A-23, 5′-CGTGTTTATGTATTATGGATT-3′ (Sigma).

### Floatation

Cell homogenate (250 μl) containing 200 μg protein was treated with 1% Triton X-100 on ice for 30 min and was adjusted to 40% iodixanol with 500 μl of OptiPrep (Invitrogen) containing 60% of iodixanol. The sample (700 μl) in TLS55 centrifuge tubes (Beckman) was overlaid with 1.2 ml of 30% iodixanol/TNE (150 mM NaCl/2 mM EDTA in 50 mM Tris⋅HCl, pH 7.4) and 0.1 ml of TNE, and centrifuged at 55,000 rpm (259,000 × *g*) for 2 h at 4 °C. Two 1-ml fractions were collected from the top. Two fractions each containing the detergent-resistant (R) and detergent-soluble (S) materials was collected. Total lipids were extracted from 700 μl of each fraction and analysed for the amount of plasmalogens and SM by liquid chromatography-electrospray ionization-tandem mass spectrometry (LC-ESI-MS/MS)^59^.

### Lipid analysis

Cells were cultured in the presence of 0.1 μCi/ml of ^14^C-Etn (Moravek). Equal aliquots (100 μg protein) of cell lysates were subjected to lipid extraction by the Bligh and Dyer method^60^. For the detection of plasmalogens, cell lysates were treated with TCA to hydrolyse vinyl-ether bond of plasmalogens prior to lipid extraction, which generates 2-acyl-GPE^2^. Lipids were analysed on TLC plates (silica gel 60, Merck) with chloroform/methanol/acetic acid solution (v/v/v: 65/25/10)^2^ or hexane/diethyl ether/acetic acid solution (v/v/v: 80/20/1.5)^16^. Total cellular plasmalogens were analysed by LC-ESI-MS/MS^59^. Distribution of plasmalogens in the outer leaflet of plasma membranes was assessed by TNBS (Wako) modification^44^. In brief, cells were metabolically labelled with ^14^C-Etn for 18 h, washed with ice-cold SHT buffer (0.25 M sucrose, 10 mM Hepes-KOH pH 8.5) containing 1 μg/ml taxol (Sigma), incubated on ice for 5 min, and further treated with 10 mM TNBS dissolved in SHT buffer on ice for 30 min in the dark. Excess TNBS was quenched for 15 min with 50 mM Tris-HCl pH 8.0. Lipids were extracted and analysed on TLC as described above. ^14^C-labelled lipids were detected by autoradiography using a FLA-5000 imaging analyser or Typhoon^TM^ FLA 9500 biomolecular imager and quantified using an image analyser software (Multi Gauge, Fuji Film).

### Immunoblot analysis

Cells were harvested in homogenizing buffer^2^. Equal aliquots of total proteins (3–5 μg) were separated by SDS-PAGE, and subjected to immunoblotting with rabbit polyclonal antibodies to Far1^4^, Pex3p^61^, Agps^2^. Rabbit antiserum to human Dhapat was raised against a carboxyl-terminal portion encompassing amino-acid residues between 343 and 680. Guinea pig anti-Pex14pC^62^, goat anti-lactate dehydrogenase antibody (LDH) (Rockland) and mouse antibodies to *β–*actin (MBL), Cav1 (Santa Cruz), Flot1 (BD Transduction Laboratories), and Tfr (Zymed Laboratories) were used. After probing with HRP-conjugated secondary antibodies, immunoblots were developed with ECL reagents (GE Healthcare), and visualized by an LAS-4000 Mini luminescent image analyser (Fuji Film). The intensity of bands was quantified by Multi Gauge software version 3.0 software (Fuji Film) within the linear range of detection. Precision Plus Protein™ All Blue Standards (BioRad) was used as standard molecular mass markers.

### Real-time PCR

Total RNA was isolated from cells using TRIzol reagent (Invitrogen) and synthesis first-strand cDNA was performed using the PrimeScript RT reagent Kit (Takara Bio). Quantitative real-time RT-PCR was performed in an Mx3000 P QPCR system (Agilent Technologies) using SYBR Premix Ex Taq II (Takara Bio). Primers used were as follows: Chinese hamster *FAR1* sense: ClFar1-287Fw. 5′-AAACACCACAAGAGCGAGTG-3′; antisense ClFar1-412Rv. 5′-CGAGTTTAGGTTGGGTGAGTTC-3′, Chinese hamster *β–actin* sense: ClbAct562Fw. 5′-ACCTGACAGACTACCTCAT-3′; antisense ClbAct630Rv. 5′-ACGCACAATTTCCCTCTC-3′, Human *FAR1* sense: HsFar1-149Fw. 5′-AGACACCACAAGAGCGAGTG-3′; antisense HsFar1-253v. 5′-CCAGTTTAGGTTGGGTGAGTTC-3′.

### Endocytosis of Tf

CHO-K1 and ZPEG251 cells were cultured in the medium containing FBS plus Dynasore for 4 h and further cultured for 1 h with medium containing Dynasore in the absence of FBS^23^. The cells were then incubated with 50 μg/ml of Tf tagged with Alexa Fluor 633 (Molecular Probes) for 15 min at 37 °C^23^. After the fixation, cells were incubated with phalloidin conjugated with Alexa Fluor 568 (Molecular Probes) according to the manufacturer’s instruction.

### Immunofluorescence Microscopy

Cells were fixed using 4% paraformaldehyde for 15 min at room temperature, permeabilised with 1% Triton X-100 for 2 min, and blocked with 1% bovine serum albumin (Nacalai Tesque) in PBS (blocking buffer) for 30 min. Cells were then incubated with indicated primary antibodies in blocking buffer for 1 h. Antigen-antibody complexes were visualized using Alexa Fluor 488-goat anti-rabbit or Alexa Fluor 647-labelled goat anti-guinea pig IgG antibody (Invitrogen). Cells were observed using a confocal laser microscope (LSM710; Carl Zeiss). Images were acquired with a ZEN2012 software (Carl Zeiss).

### Data presentation

Expression level of Far1 was normalized by the expressed level of *β–*actin in each sample. Quantitative data were shown as mean ± S.D from at least three independent experiments. Statistical significance was determined by Student’s *t*-test, or ANOVA with Dunnett’s, Tukey, or Tukey-Kramer post hoc test using R software (http://www.r-project.org).

## Additional Information

**How to cite this article:** Honsho, M. *et al*. Plasmalogen biosynthesis is spatiotemporally regulated by sensing plasmalogens in the inner leaflet of plasma membranes. *Sci. Rep.* **7**, 43936; doi: 10.1038/srep43936 (2017).

**Publisher's note:** Springer Nature remains neutral with regard to jurisdictional claims in published maps and institutional affiliations.

## Supplementary Material

Supplementary Information
