# Supplementary material for: Plasmalogen biosynthesis is spatiotemporally regulated by sensing plasmalogens in the inner leaflet of plasma membranes
Source: Sci Rep. 2017 Mar 8;7:43936. doi: 10.1038/srep43936 (PMC5341075; doi:10.1038/srep43936)

Supplementary Information

Plasmalogen biosynthesis is spatiotemporally regulated by sensing plasmalogens in the inner leaflet of plasma membranes

Masanori Honsho, Yuichi Abe, and Yukio Fujiki*

Supplementary Fig. S1

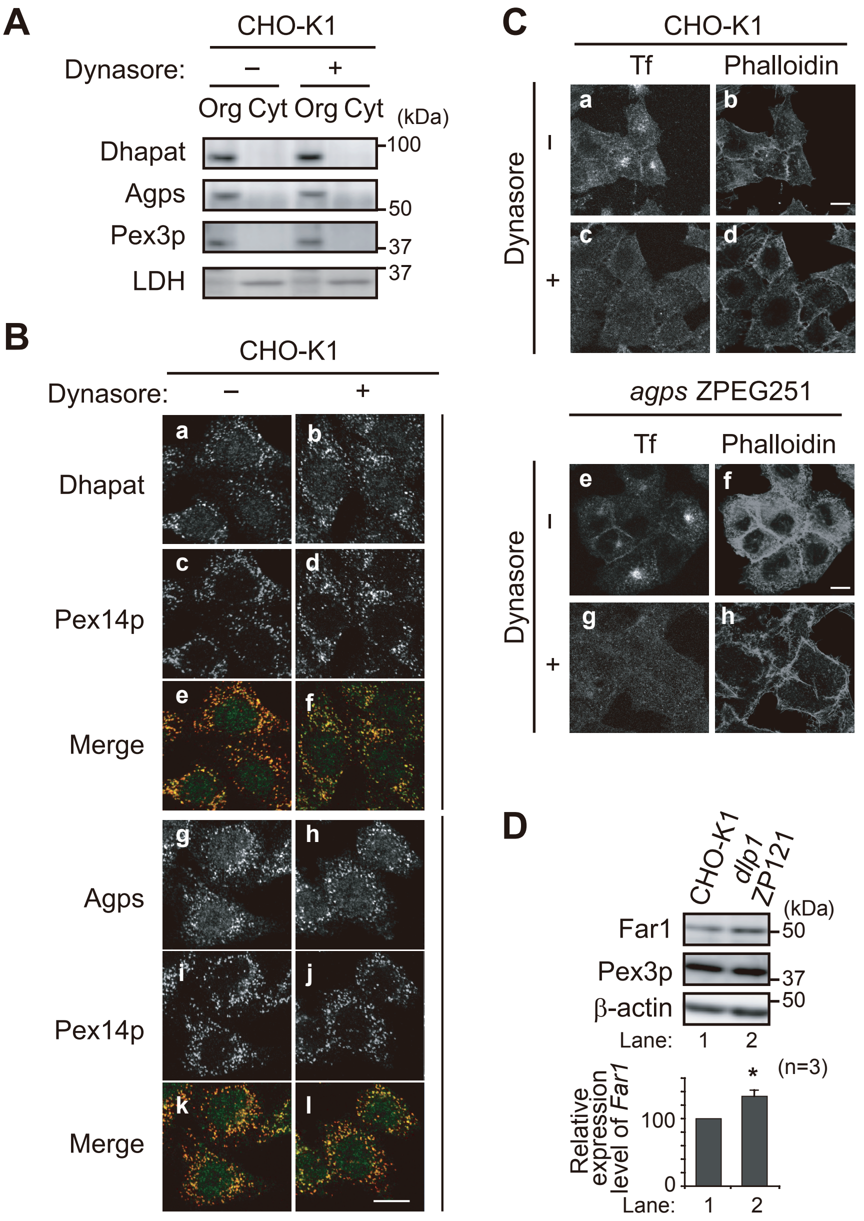

Figure S1.

Intracellular localisation of peroxisome matrix proteins and expression level of Far1 in *dlp1* CHO mutant ZP121.

(***A***) Organelle (Org) and cytosolic (Cyt) fractions were prepared from CHO-K1 cultured in the presence (+) or absence (–) of Dynasore, and assessed by immunoblotting with antibodies against Dhapat, Agps, Pex3p, and lactate dehydrogenase (LDH). (***B***) CHO-K1 cells were cultured in the presence (*b, d, f, h, j,* and *l*) or absence (*a, c, e, g, i*, and *k*) of Dynasore, and intracellular localisation of Dhapat (*upper panels, a* and *b*), Agps (*lower panels*, *g* and *h*), and Pex14p (*c, d, i,* and *j*), a peroxisomal membrane protein, was assessed by staining with respective antibodies. Merged views are shown (*e, f, k,* and *l*). Scale bar, 10 µm. (***C***) CHO-K1 and ZPEG251 were cultured in the presence (*c, d, g,* and *h*) or absence (*a, b, e,* and *f*) of Dynasore, and examined for the endocytosis of Tf conjugated with Alexa Fluor 633 (*a, c, e,* and *g*). Cells were stained with phalloidin conjugated with Alexa Fluor 568 (*b, d, f,* and *h*). Scale bar, 10 µm. (***D***) Expression of Far1 in CHO-K1 and *dlp1* ZP121 was assessed by immunoblotting. β-actin was used as a loading control. Relative expression levels of Far1 in ZP121 (lane 2) were represented by taking 100 that in CHO-K1 (lane 1). *p<0.05; *t* test *versus* CHO-K1. Data of unprocessed original blots are available in Supplementary Fig. S2.

Supplementary Fig. S2

Data represented in the main Figures are marked with dashed line boxes and shown by the lane numbers.

Fig. 1A

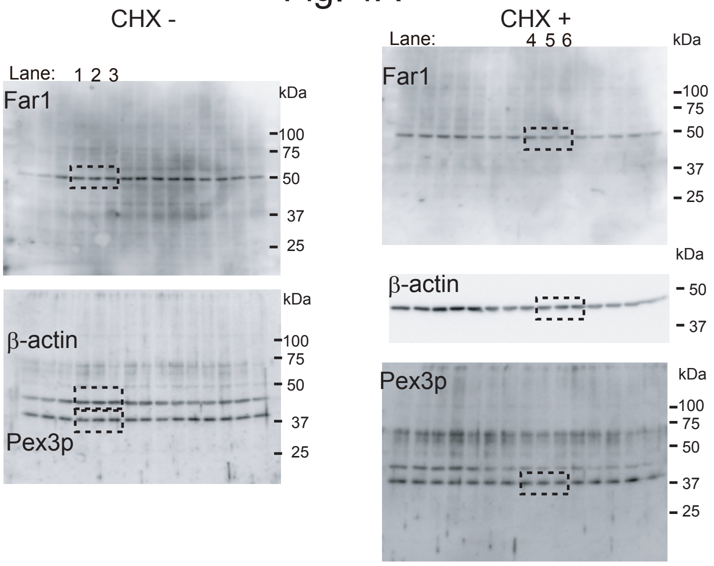

Fig. 1B

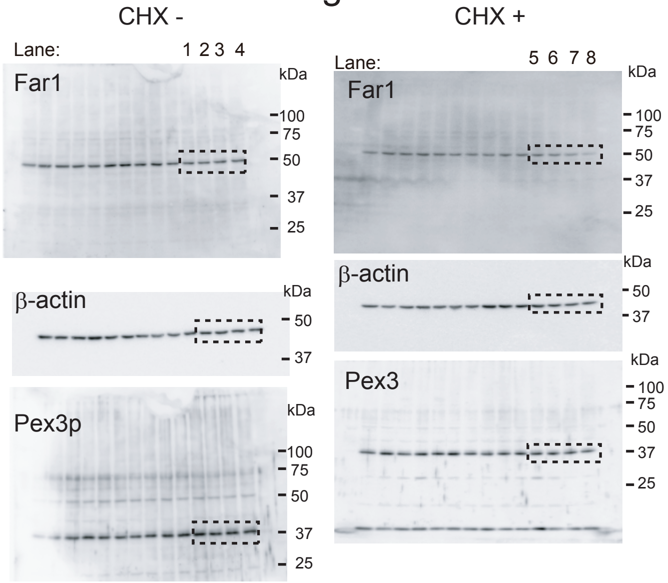

Fig. 3A

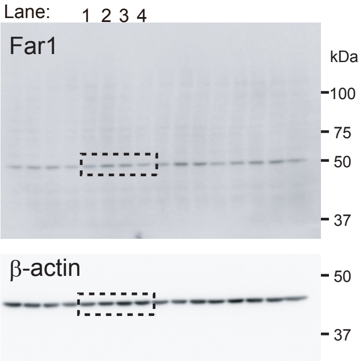

Fig. 3B

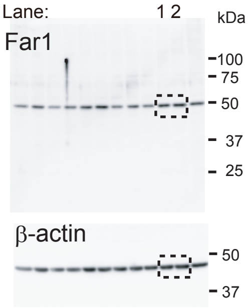

Fig. 3F

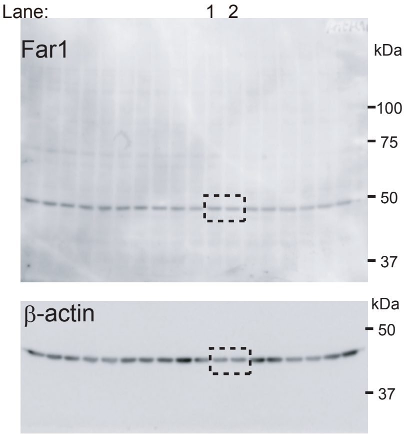

Fig. 4A

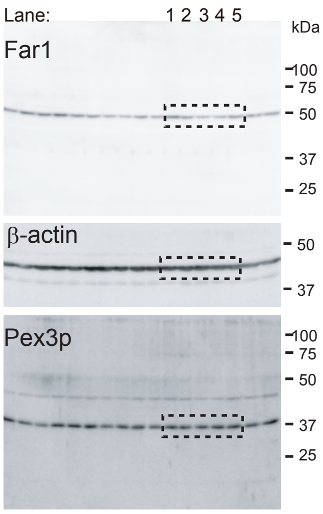

Fig. 5A

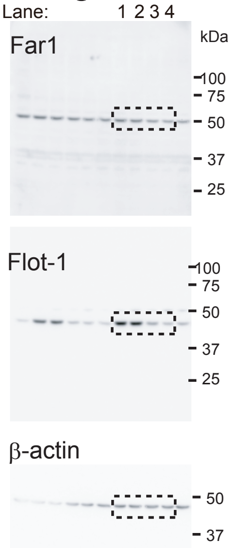

Supplementary Fig. S2 (continued)

Data represented in the main Figures are marked with dashed line boxes and shown by the lane numbers.

Fig. 6A

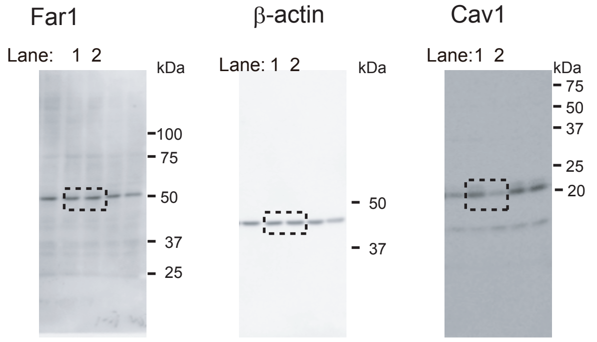

Fig. 6B

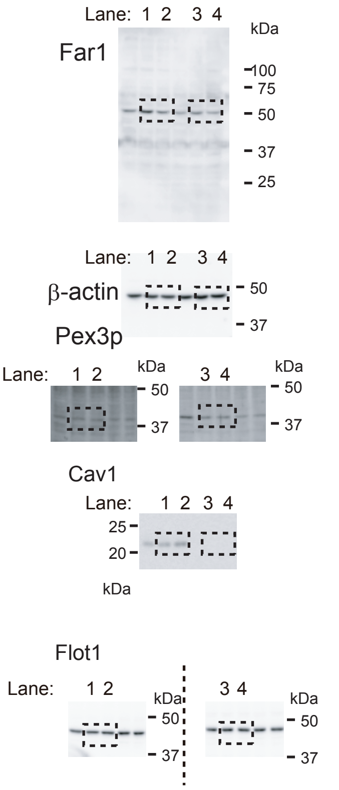

Fig. 7A

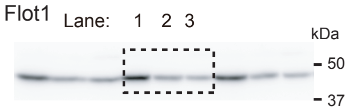

Fig. 7B

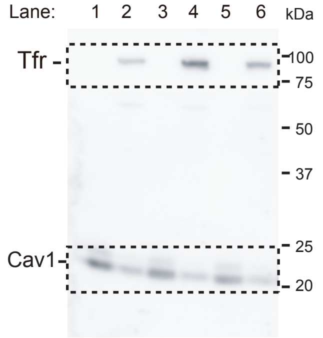

Fig. 7E

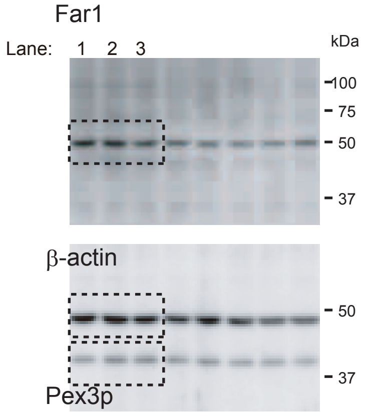

Fig. 8A

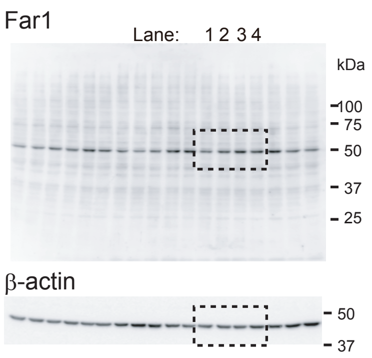

Fig. S1A

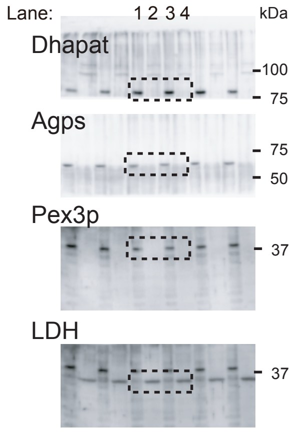

Fig. S1D

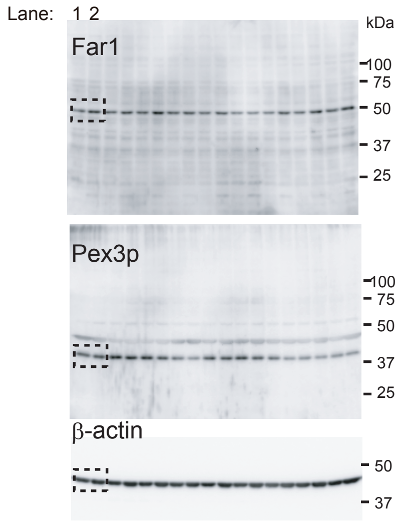

Supplement: Supplementary Information [file srep43936-s1.pdf]
